# The Strychnine Cure for Drunkenness

**Published:** 1892-02

**Authors:** 


### THE STRYCHNINE CURE FOR DRUNKENNESS.

MR EDITOR: I am sure you have heard of the celebrated physician who told his patient he could do nothing for his ailments, but that he could throw him into fits, and that he was death on fits.

Well, sir, you will find in the December, 1891, *Medical Advance* an editorial based upon the idea of the physician mentioned above. In this article we are told that, "If we can destroy the appetite for stimulants by the Strychnine cure [which consists of one or two subcutaneous injections daily of solution of Strychnine, two grains to the ounce], we can then treat the patient with constitutional remedies, and by removing the taint by antipsorics prevent a relapse."

Do you not think the writer of the article should make application for junior membership in the International Hahnemannian Association so that he might be taught what Homœopathy is?

AN ANXIOUS INQUIRER.

